# Cuproptosis gene‐related, neural network‐based prognosis prediction and drug‐target prediction for KIRC

**DOI:** 10.1002/cam4.6763

**Published:** 2023-12-22

**Authors:** Yixin Liu, Yuan Shao, Zezhou Hao, Xuanzi Lei, Pengchen Liang, Qing Chang, Xianjin Wang

**Affiliations:** ^1^ Department of Surgery, Shanghai Key Laboratory of Gastric Neoplasms Shanghai Institute of Digestive Surgery, Ruijin Hospital Affiliated to Shanghai Jiao Tong University School of Medicine Shanghai China; ^2^ School of Health Science and Engineering University of Shanghai for Science and Technology Shanghai China; ^3^ Department of Urology Ruijin Hospital Affiliated to Shanghai Jiao Tong University School of Medicine Shanghai China; ^4^ Graduate School Shanghai University of Traditional Chinese Medicine Shanghai China; ^5^ School of Microelectronics Shanghai University Shanghai China

**Keywords:** cuproptosis‐related genes, drug‐target prediction, KIRC, neural network, prognosis prediction

## Abstract

**Background:**

Kidney renal clear cell carcinoma (KIRC), as a common case in renal cell carcinoma (RCC), has the risk of postoperative recurrence, thus its prognosis is poor and its prognostic markers are usually based on imaging methods, which have the problem of low specificity. In addition, cuproptosis, as a novel mode of cell death, has been used as a biomarker to predict disease in many cancers in recent years, which also provides an important basis for prognostic prediction in KIRC. For postoperative patients with KIRC, an important means of preventing disease recurrence is pharmacological treatment, and thus matching the appropriate drug to the specific patient's target is also particularly important. With the development of neural networks, their predictive performance in the field of medical big data has surpassed that of traditional methods, and this also applies to the field of prognosis prediction and drug‐target prediction.

**Objective:**

The purpose of this study is to screen for cuproptosis genes related to the prognosis of KIRC and to establish a deep neural network (DNN) model for patient risk prediction, while also developing a personalized nomogram model for predicting patient survival. In addition, sensitivity drugs for KIRC were screened, and a graph neural network (GNN) model was established to predict the targets of the drugs, in order to discover potential drug action sites and provide new treatment ideas for KIRC.

**Methods:**

We used the Cancer Genome Atlas (TCGA) database, International Cancer Genome Consortium (ICGC) database, and DrugBank database for our study. Differentially expressed genes (DEGs) were screened using TCGA data, and then a DNN‐based risk prediction model was built and validated using ICGC data. Subsequently, the differences between high‐ and low‐risk groups were analyzed and KIRC‐sensitive drugs were screened, and finally a GNN model was trained using DrugBank data to predict the relevant targets of these drugs.

**Results:**

A prognostic model was built by screening 10 significantly different cuproptosis‐related genes, the model had an AUC of 0.739 on the training set (TCGA data) and an AUC of 0.707 on the validation set (ICGC data), which demonstrated a good predictive performance. Based on the prognostic model in this paper, patients were also classified into high‐ and low‐risk groups, and functional analyses were performed. In addition, 251 drugs were screened for sensitivity, and four drugs were ultimately found to have high sensitivity, with 5‐Fluorouracil having the best inhibitory effect, and subsequently their corresponding targets were also predicted by GraphSAGE, with the most prominent targets including Cytochrome P450 2D6, UDP‐glucuronosyltransferase 1A, and Proto‐oncogene tyrosine‐protein kinase receptor Ret. Notably, the average accuracy of GraphSAGE was 0.817 ± 0.013, which was higher than that of GAT and GTN.

**Conclusion:**

Our KIRC risk prediction model, constructed using 10 cuproptosis‐related genes, had good independent prognostic ability. In addition, we screened four highly sensitive drugs and predicted relevant targets for these four drugs that might treat KIRC. Finally, literature research revealed that four drug‐target interactions have been demonstrated in previous studies and the remaining targets are potential sites of drug action for future research.

## Graphical abstract

Deep neural network models were established using kidney renal clear cell carcinoma (KIRC) data from TCGA and validated using International Cancer Genome Consortium data to evaluate the performance. Then, various differential analyses were performed on high‐ and low‐risk groups, personalized clinical decision models were established, and KIRC high sensitivity drugs were screened. Finally, KIRC graph data modeling was carried out using DrugBank's KIRC‐related drug and target data, and target prediction of KIRC high sensitivity drugs was performed based on GraphSAGE.

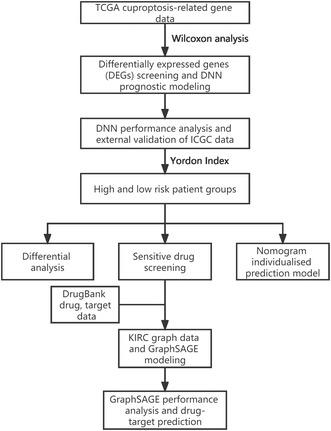

## 1 BACKGROUND

Renal cell carcinoma (RCC) is a stealthy tumor that ranks sixth in incidence among malignant tumors in adult males and tenth among malignant tumors in females.^1^ As one of the seven most common malignant tumors worldwide, RCC accounts for approximately 2% of cancer diagnoses and deaths globally.^2^ According to the 2018 GLOBOCAN database, there were 403,262 new cases of RCC and 175,098 deaths, accounting for 2.5% of all cancer types.^3^ Kidney renal clear cell carcinoma (KIRC) accounts for 80% of RCC cases, KIRC is also a highly immune‐infiltrating tumor and one of the first malignancies to respond to immunotherapy.^4^ The tumor suppressor gene VHL mutation causes the loss of its ubiquitinated degradation of HIF‐1α, making stabilizing the level of HIF‐1α a key factor in treating cancer.^5^ Due to this gene mutation, clear cell carcinoma often metastasizes, making it an important cause of death among RCC patients.^6^ In clinical practice, most patients with KIRC who receive chemotherapy, radiotherapy, or targeted therapy have more significant side effects, so surgical resection is still the main treatment option, but the recurrence rate of about 30% after surgery is a factor in the poor prognosis of patients.^7^ Currently, commonly used monitoring methods include blood biomarker detection and imaging methods, but they still have shortcomings such as low sensitivity and specificity.^8^ Therefore, new molecular markers are needed to establish new prognostic models and provide new reference methods for KIRC prognostic monitoring.

Copper ions are a “double‐edged sword.” The initial understanding of copper ions was that they are important kinases in the metabolism, organic energy conversion, and aerobic oxidation of biological cells. At physiological concentrations, they play an important role in the electron transport chain in mitochondria,^9^ but at high concentrations, they can produce cell toxicity. Tsvetkov et al. first reported a copper ion‐mediated programed cell death (PCD) pattern and named it cuproptosis.^10^ Mechanistically, it is dependent on copper ion concentration and differs from known PCD, such as necrosis, apoptosis, pyroptosis, and ferroptosis, mainly acting on lipoylated components of the tricarboxylic acid (TCA) cycle to regulate cell death. Cuproptosis, a newly discovered mode of cell death, is being extensively studied and it was found that high expression of copper transporter 1 (CTR1) in tumors was independently associated with low overall survival (OS) in KIRC.^11^ Moreover, research has found that endoplasmic reticulum (ER) stress in RCC is caused by cuprous oxide nanoparticles interfering with copper transport and promoting intracellular calcium and reactive oxygen species (ROS) accumulation.^12^ It can be seen that KIRC is closely related to the regulation of cuproptosis genes and cuproptosis genes can be used as new molecular markers for KIRC.

In the field of disease prediction, prognostic models are widely used to perform patient risk prediction. Chen et al. used necroptosis‐related genes to establish a prognostic model for KIRC, and the model had an AUC of 0.707, which was able to better predict the risk of the patients.^13^ Zhang et al. used cuproptosis‐related genes to establish a prognostic model for hepatocellular carcinoma, and the model's AUC on the TCGA dataset was 0.63.^14^ Although all of these studies were able to predict the risk of patients better, they all built cox risk prediction models, which are only capable of linear analysis. While in reality, risk prediction is likely to be nonlinear.

Neural networks are a technique in the field of machine learning, which is an extension of perceptron and can analyze nonlinear relationships well. It consist of input layer, hidden layer, and output layer. During the training of neural networks, each hidden layer continuously adjusts the weights of the previous layer to update the iterative data, in order to minimize bias. Increasing the number of hidden layers can make neural networks more efficient in handling massive data.^15^ With the development of artificial intelligence in the medical field, this technology has been applied to various medical research and has demonstrated superior learning ability, for example in prognosis.^16^ In the field of drug research, most of the cutting‐edge work has been carried out based on molecular simulation and computer docking,^17^, ^18^, ^19^, ^20^ which allows for effective prospective studies of drug action mechanisms by simulating the docking process of individual drug molecules with protein targets. Although the simulated docking approach has achieved excellent success in drug development for most diseases, this is only for individual drugs or targets and is not applicable to batch screening. Graph neural network (GNN) models, a branch of neural networks, have become an emerging force in drug discovery and development in recent years.^21^ Using its unique unstructured analysis method, GNN models can predict the relationship between drugs and corresponding targets, diseases, and other related entities, and ultimately select suitable drugs or targets. In addition, the advantages of neural networks in big data analysis enable GNNs to screen multiple drugs or targets at the same time, thus acting as a high‐throughput screen.

As the cuproptosis gene was found to potentially serve as a biomarker for KIRC and the mechanism of association between KIRC prognosis and the cuproptosis gene is unclear, this study will predict the survival probability of patients with KIRC by establishing a prognostic model based on the cuproptosis gene, which will provide a valuable reference for subsequent clinical decision‐making. In addition, we will screen highly sensitive drugs for the treatment of KIRC and have predicted the targets of these highly sensitive drugs using the GNN method, providing new insights and ideas for the treatment of KIRC with targeted drugs.

## 2 DATA AND METHODS

### 2.1 Gene data

**FIGURE 1:**
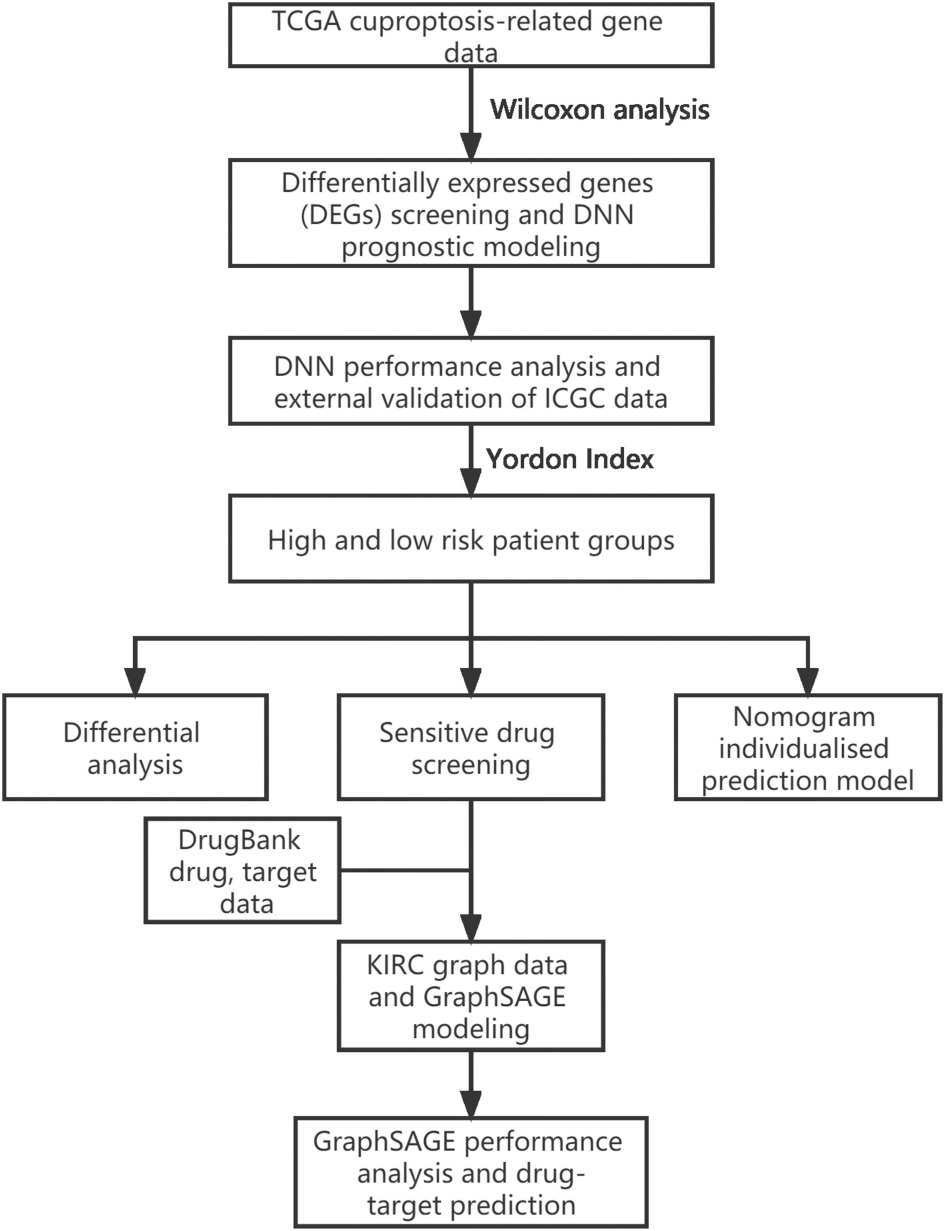
A flowchart for KIRC prognosis and drug‐target prediction based on artificial intelligence. DNN models were established using KIRC data from TCGA and validated using ICGC data to evaluate the performance. Then, various differential analyses were performed on high‐ and low‐risk groups, personalized clinical decision models were established, and KIRC high sensitivity drugs were screened. Finally, KIRC graph data modeling was carried out using DrugBank's KIRC‐related drug and target data, and target prediction of KIRC high sensitivity drugs was performed based on GraphSAGE.

We obtained sequencing and clinical data for KIRC from the TCGA database (version 33.0; https://portal.gdc.cancer.gov/projects/), including sequencing data from 613 samples from 537 patients, which included 72 normal samples and 541 tumor samples, and clinical features such as age, gender, stage, grade, and T, M, and N staging. As the N staging feature was unevenly distributed and mostly unknown, this clinical feature was excluded from subsequent experiments. We used the TCGA data as the internal dataset, and for external validation, we also obtained sequencing and clinical data for KIRC from the ICGC database (release 28; https://dcc.icgc.org/). For the ICGC data, we only selected available tumor samples with corresponding gene expression data, which included sequencing data from 518 samples from 518 patients and clinical features such as age and gender. We obtained 12 cuproptosis‐related genes from Tsvetkov et al.'s study, including FDX1, LIPT1, LIAS, DLD, DBT, DLST, DLAT, PDHA1, PDHB, SLC31A1, ATP7A, and ATP7B.^10^ In addition, we analyzed the progression‐free survival (PFS) and tumor mutation burden (TMB) data for KIRC from the TCGA dataset. The flowchart of our study is shown in Figure 1.

### 2.2 Graph data

We searched for KIRC‐related targets and corresponding drugs in the DrugBank database (version 5.1.10; https://go.drugbank.com/) as our training data. After filtering and deduplication, we obtained 11 small molecule drugs and 48 targets related to KIRC. Due to the limited amount of KIRC‐related data, we supplemented the target data with targets that have a similarity of over 90% with KIRC targets, resulting in a total of 341 targets. We also supplemented the drug data with four drugs selected by bioinformatics methods, resulting in a total of 15 drugs. Finally, based on these 15 drugs and 341 targets, we constructed a KIRC graph data consisting of 1250 nodes and 405,226 edges. For each node, we only set a connection relationship between one drug and one target, and then determine whether to label the node as 1 based on the interaction between the drug and target. For node features, we initially obtained 9975‐dimensional features for drugs and targets, and reduced the feature dimension to 249 using feature selection methods. Edges were established based on whether there is a connection between two drugs or two targets, and if there is a connection between both, the edge feature is set to 1; if only one exists, the edge feature is set to 0.5, and if there is no connection, the edge feature is set to 0.

### 2.3 Identification methods for differentially expressed cuproptosis‐related genes and construction of deep neural network prognostic model

**FIGURE 2:**
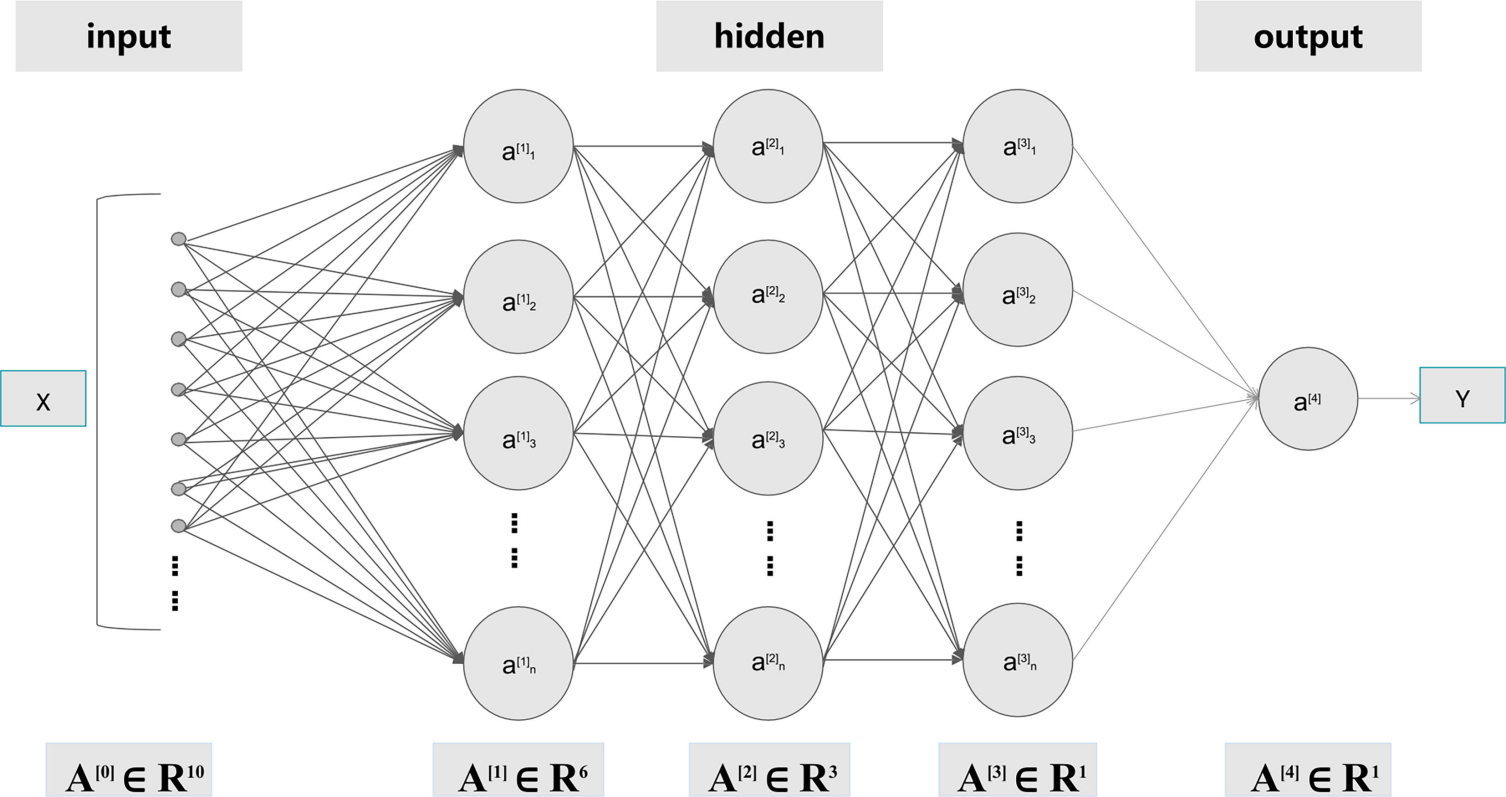
Architecture diagram of the DNN model. The number of neurons in each layer is 6, 3, and 1, respectively.

We conducted a Wilcoxon test for differential analysis and selected 10 cuproptosis‐related genes with *p <* 0.05 and FDR < 0.05 as the differentially expressed genes (DEGs). Considering the sequencing errors between TCGA and ICGC data, we standardized the data separately using 0–1 normalization. We established a deep neural network (DNN) model based on 10 DEGs and TCGA data, with the model architecture that includes one input layer, three hidden layers, and one output layer. Our model was trained using the TensorFlow framework, and employed a Tanh activation function and stochastic gradient descent optimizer, as well as using a hybrid L1 and L2 regularization method and a dropout method to prevent overfitting. In addition, we used a Bayesian optimization tuning method to fit the most suitable hyperparameters for the model, including learning rate of 0.9, learning rate decay of 0.9999, L1 regularization of 0.0005144577800049358, L2 regularization of 0.00010446573118591082, and dropout rate of 0.1. Finally, we assessed the training status of the model through the concordance index (*C*‐index) curve and the loss function curve. The model architecture is illustrated in Figure 2.

### 2.4 High‐ and low‐risk grouping and model performance evaluation

We evaluated the predictive accuracy of the model by plotting receiver operating characteristic (ROC) curves for 1‐, 3‐, and 5‐year periods, and compared the constructed prognostic model with the traditional Cox proportional hazards (Cox) model. Subsequently, the optimal cutoff value on the ROC curve in the first year was identified using the Youden index, and the samples were divided into high‐ and low‐risk groups, with principal component analysis (PCA) used for evaluation. We also analyzed the survival differences between groups using Kaplan–Meier survival curves, and finally assessed the model's independent prognostic ability through univariate and multivariate Cox analysis.

### 2.5 Analysis of differences between high‐ and low‐risk groups

#### 2.5.1 Aspects of clinical features

We combined PFS data to assess whether there was a significant difference in progression‐free survival between the high‐ and low‐risk groups, and subsequently generated a heatmap of the groups and clinical features. It is worth mentioning that we divided the clinical feature stage into early stages I‐II and late stages III‐IV, and investigated the survival differences between high‐ and low‐risk groups.

#### 2.5.2 Aspects of functionality

In order to investigate differences between the high‐ and low‐risk groups at the genetic, immune and tumor mutation levels, we conducted functional analysis. At the gene level, we performed GO and KEGG enrichment analyses to investigate differences in gene pathways between high‐ and low‐risk groups. At the immune level, we analyzed the immune microenvironment using single sample gene set enrichment analysis (ssGSEA), with a total of 16 types of immune cells analyzed. For tumor mutations, we used the Maftools package in R to perform tumor mutation burden (TMB) analysis, calculating the TMB score of each sample and mutation frequency of each gene in the sample, and then dividing the samples into high TMB and low TMB groups. We analyzed the top 15 genes with the highest mutation frequency, and combined the TMB results with the DNN grouping results to analyze overall survival.

### 2.6 Construction of an individualized clinical decision model

In consideration of the individual differences in survival time among different samples, we established a personalized clinical decision model, a nomogram model. By analyzing the *C*‐index of DNN's prediction results and clinical features over 0–10 years, we finally selected DNN's prediction results and clinical features age, stage, grade, T stage, and M stage to jointly build the model. We used calibration curves to assess the reliability of the model's results at first, third, and fifth years, and used a high‐risk sample and a low‐risk sample to visually display the model.

### 2.7 Screening of sensitive anticancer drugs

We used the pRRophetic package in R to analyze the sensitivity differences of 251 drugs between high‐ and low‐risk groups, and screened potential sensitivity drugs for the treatment of high‐risk KIRC patients based on IC50 values. The screening criteria require that the IC50 value of the high‐risk group is lower than that of the low‐risk group and the P value is within a given range. For the sensitivity drugs selected, we designed CCK‐8 assay and colony formation assay to verify their effectiveness. The main experimental steps are as follows:

1. Steps of the CCK‐8 assay
  - Take well‐grown 786‐O and Caki‐1 cells, digest and centrifuge them to prepare a single‐cell suspension, and adjust the cell concentration to 1 × 10^5^ cells/mL.
  - Add 100 μL of cell suspension per well to a 96‐well plate, divide into experimental groups, set up three replicates per group, and incubate in a 37°C culture incubator with 5% CO_2_ for 24 h.
  - Treat the two types of cells separately in different experimental groups for 0, 24, 48, and 72 hr. After treatment, add 10 μL of CCK‐8 solution to each well and incubate in the culture incubator for 4 h. Measure the optical density (OD) value of each well with an microplate reader for subsequent analysis.
2. Steps of the colony formation assay
  - After trypsin digestion, resuspend logarithmically growing 786‐O and Caki‐1 cells in culture medium, and count the cells. Seed 1000 cells per well in a 6‐well plate, and incubate in a 37°C culture incubator with 5% CO_2_ for 24 h.
  - Treat the two types of cells separately in different experimental groups for 14 days. During the culture period, change the medium every 3 days and observe the cell status.
  - After cloning, wash once with PBS and add 1 mL of 4% paraformaldehyde to each well, fix for 30–60 min. Wash one to three times with PBS, then add 1 mL of crystal violet staining solution to each well for 10–20 min.
  - Wash the cells three times with PBS and air dry. Then take a photo of the entire 6‐well plate.

### 2.8 Graph neural network

As our research requires the prediction of targets for four drugs selected by bioinformatics methods that have not appeared in the training data, we chose GraphSAGE,^22^ which is more suitable for inductive learning, as our research model. The main equation of GraphSAGE is as follows:

(1) $$h_{N\left(v\right)}^{k}\leftarrow {\text{AGGREGATE}}_{k}\left(\left\{h_{u}^{k-1},\forall u\in N\left(v\right)\right\}\right)$$

(2) $$h_{v}^{k}\leftarrow \sigma \left(W^{k}\cdot \text{CONCAT}\left(h_{v}^{k-1},h_{N\left(v\right)}^{k}\right)\right)$$

In the *k* layer of graph convolutional layer, the mini‐batch sampling is first applied to the neighbor nodes $h_{u}^{k-1}$ of the current node $h_{N\left(v\right)}^{k}$, and multiple subgraphs are obtained. Then, the feature vectors of the neighbor nodes $h_{u}^{k-1}$ of the current node $h_{N\left(v\right)}^{k}$ on the subgraph are aggregated to obtain the neighbor feature representation of the current node $h_{N\left(v\right)}^{k}$. Then, the current node $h_{N\left(v\right)}^{k}$ is concatenated with the neighbor feature representation, and finally, the feature representation $h_{v}^{k}$ of the *k* graph convolutional layer on a single subgraph is obtained. By performing graph convolutional operations with *k* = 3 layers, the model can learn the node information of the entire graph.

We chose to use the average method to aggregate node feature vectors. The main equation is as follows:

(3) $$h_{v}^{k}\leftarrow \sigma \left(W\cdot \text{MEAN}\left(\left\{h_{v}^{k-1}\right\}\cup \left\{h_{u}^{k-1},\forall u\in N\left(v\right)\right\}\right)\right.$$

In each aggregation operation, the model applies element‐wise averaging to the feature representation obtained by concatenating the current node with the neighbor nodes, applies a nonlinear transformation to the result, and finally produces the *k* layer feature representation $h_{v}^{k}$ of the current node. Then, the *k* layer feature representation $h_{v}^{k}$ of the current node is used as the feature vector of the node at *k* + 1 layer, and the node feature aggregation continues.

The training mode of GraphSAGE is not to learn the features of each node, but to learn how to aggregate the feature representations of multiple nodes. After multiple training iterations, this inductive learning method can generalize the way each mini‐batch sampled subgraph is learned. Therefore, when new nodes are added, this inductive learning method can generate new feature representations for completely unseen nodes without retraining the entire graph. Transductive GNN models cannot achieve this, because they need to generate a new graph for the new node and train the adjacency matrix of the new graph to update the weight.

### 2.9 Statistical methods

This study employed R language and RStudio software for data analysis and generating statistical charts. For Cox model, selective modeling was based on Lasso regression. In the independence analysis, *p* values for univariate Cox and multivariate Cox were set as less than 0.001. For sensitivity drug screening, *p* value was set as less than 10^−10^.

## 3 RESULTS

### 3.1 Differentially expressed cuproptosis‐related genes and training results of DNN prognostic model

**FIGURE 3:**
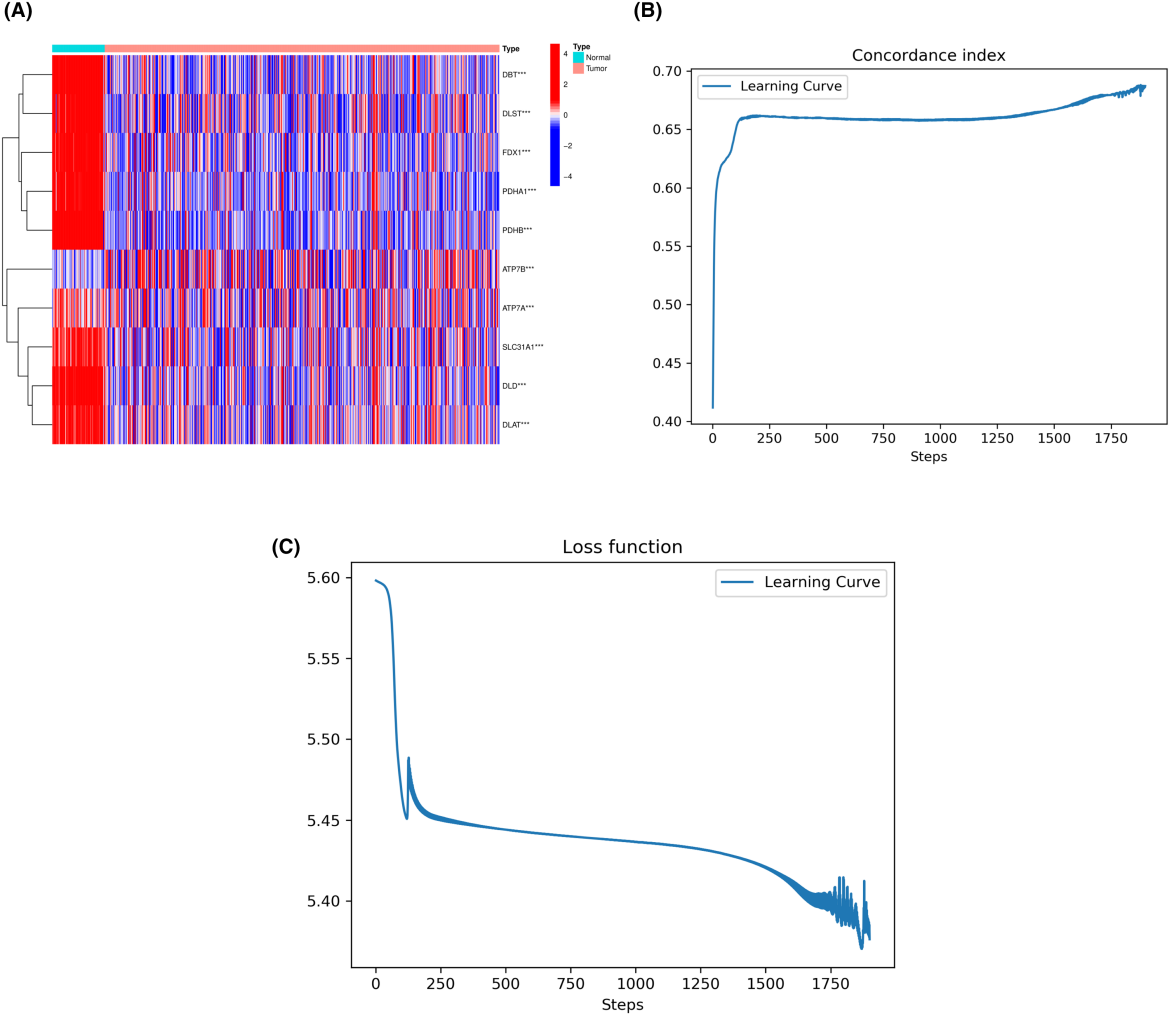
Selection of differentially expressed cuproptosis‐related genes and training status of DNN model. (A) 10 differentially expressed cuproptosis‐related genes. (B) *C*‐index plot of the DNN model. (C) Loss function plot of the DNN model.

Based on TCGA data, we performed Wilcoxon test to analyze 12 cuproptosis‐related genes and identified 10 DEGs. We also generated a gene expression heatmap, as shown in Figure 3A. To better understand the relationship between cuproptosis‐related genes and patient prognosis, we filtered and processed the tumor data from TCGA, removed five samples lacking survival status features, and standardized the remaining 532 samples using the 0‐1 method. We then built a DNN model and observed the training progress of the model using the *C*‐index curve and the loss function curve. As shown in Figure 3B,C, both curves converged with increasing training iterations.

### 3.2 Evaluation of DNN prognostic model and results of external validation on the ICGC database

**FIGURE 4:**
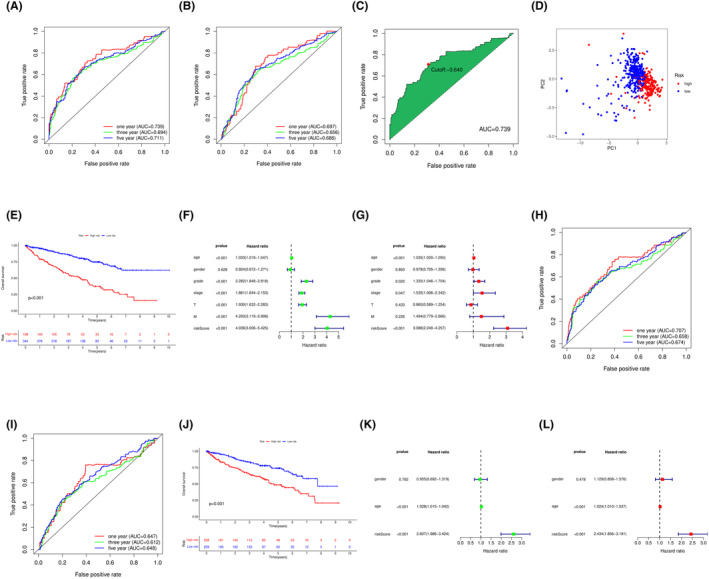
Performance analysis of DNN model. (A–G) show the training results on TCGA data, and (H–L) show the validation results on ICGC data. (A) and (H) are ROC curves of the DNN model. (B) and (I) are ROC curves of the Cox model. (C) is the optimal threshold (the Youden index) calculated based on the ROC curve on TCGA data. (D) shows the PCA plot of the DNN model grouping. (E) and (J) are survival curves based on high and low risk. (F) and (K) are univariate Cox proportional hazards models for assessing independent factors. (G) and (L) are multivariate Cox proportional hazards models for assessing independent factors.

We plotted ROC curves to evaluate the performance of our model, as shown in Figure 4A, and compared it with the traditional Cox model, as shown in Figure 4B. It can be seen that the AUC values of our DNN model were higher than those of the traditional Cox model in the first, third, and fifth years. We determined the optimal cutoff value to be −0.64 using the Youden index, as shown in Figure 4C, and divided the samples into high‐ and low‐risk groups based on this value. Figure 4D shows the PCA‐based separation of the high‐ and low‐risk groups. In addition, to study the differences in survival time between the high‐ and low‐risk groups, we also plotted survival curves, as shown in Figure 4E. The high‐risk group had a much shorter survival time than the low‐risk group (*p* < 0.001), and most of the high‐risk group also had a much shorter time to death than the low‐risk group. Cox analysis, as shown in Figure 4F,G, revealed that the DNN model had good independence. We selected the ICGC database as an external validation set, standardized the ICGC data to 0–1 and input it into the DNN model for risk score calculation, and divided the samples into high‐ and low‐risk groups according to the optimal cutoff value. We also plotted ROC curves to evaluate and compare the performance of the model, as shown in Figure 4H,I, and found that the performance of the DNN model was superior to that of the Cox model. Finally, the survival curves of the external validation set, as shown in Figure 4J, revealed significant differences between the high‐ and low‐risk groups (*p* < 0.001), and the difference analysis in Figure 4K,L showed that the DNN model also had good independence in the external validation set.

### 3.3 Relationship between risk grouping and clinical features

**FIGURE 5:**
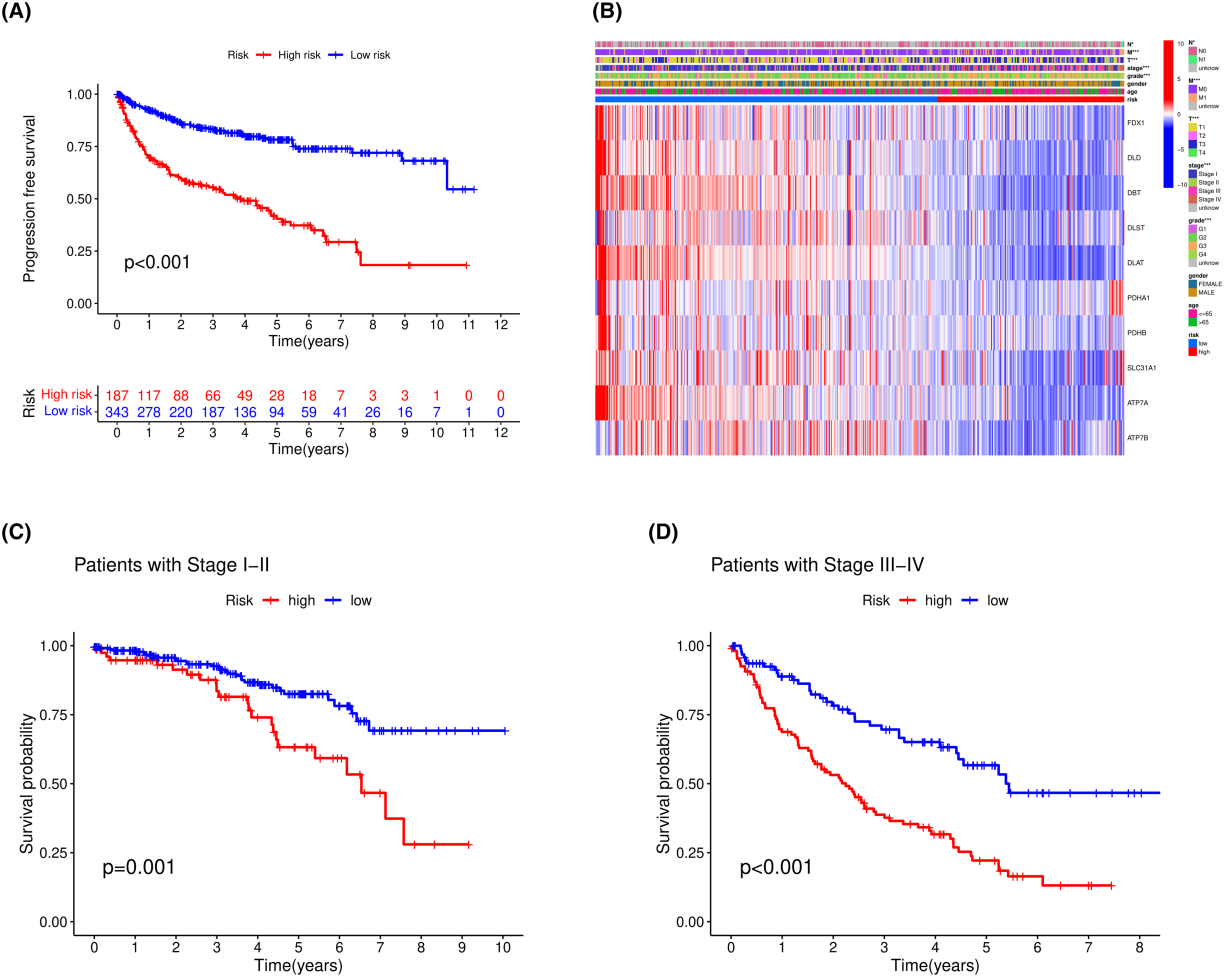
Differences in clinical features between high‐ and low‐risk groups. (A) shows the survival curve based on PFS. (B) is a heatmap of the differences between clinical features and DNN model prediction results. (C) and (D) show the survival curves of patients in the early (I–II) and late (III–IV) clinical stages, respectively.

To investigate whether there were differences in PFS between high‐ and low‐risk groups, we plotted the survival curve based on PFS data, as shown in Figure 5A, and found a significant difference between the groups (*p* < 0.001). We further examined the relationship between the risk groups and clinical features by creating a heatmap of the differences between the groups and clinical features, as shown in Figure 5B. Significant differences were observed in stage (*p* < 0.001), grade (*p* < 0.001), T stage (*p* < 0.001), and M stage (*p* < 0.001) between the groups. We then conducted a further analysis of the stage feature to determine whether the model was effective for clinical use, as shown in Figure 5C,D). The results showed that the model had good grouping effects, regardless of whether it was used for early‐stage (I‐II) or late‐stage (III‐IV) patients (*p*_I−II_ = 0.001, *p*_III−IV_ < 0.001).

### 3.4 Functional analysis of high‐ and low‐risk grouping

**FIGURE 6:**
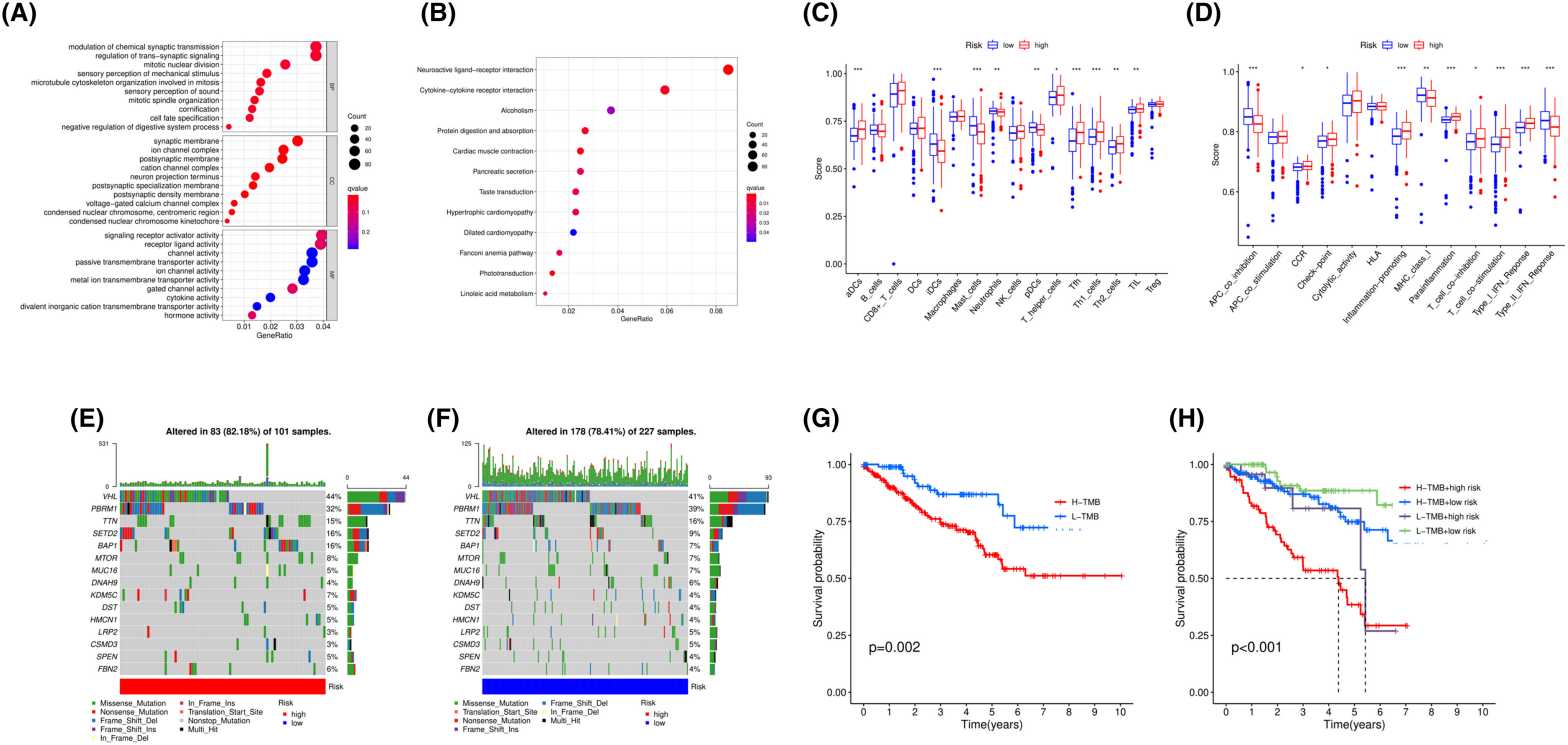
Functional differences between high‐ and low‐risk groups. (A) and (B) represent the results of GO enrichment analysis and KEGG enrichment analysis, respectively. (C) and (D) present the differential analysis of 16 immune cells and 15 immune pathways using the ssGSEA method. (E) and (F) show the waterfall plots of high and low TMB samples based on the top 15 genes with the highest mutation frequencies, respectively. (G) depicts the survival curve based on high and low TMB, while (H) combines the survival curves of high/low TMB and high‐/low‐risk groups.

To investigate the differences in gene pathways between high‐ and low‐risk groups, we conducted GO and KEGG enrichment analysis based on TCGA data. First, using Wilcoxon test analysis and filtering criteria of FDR < 0.05 and |log2(FC)| ≥ 1, we screened out 5129 DEGs, including 1608 downregulated genes and 3521 upregulated genes. We performed enrichment analysis on the screened DEGs, as shown in Figure 6A,B, and found significant differences in modulating chemical synaptic transmission, signaling receptor activator activity, and neuroactive ligand‐receptor interaction, among others. To investigate the functional differences in the immune microenvironment between high‐ and low‐risk groups, we used the ssGSEA method to analyze the differences in 16 immune cells and immune pathways, as shown in Figure 6C,D, and found significant differences in immune cells such as *aDCs*, *iDCs*, *Mast_cells*, *Tfh*, and *Th1_cells*, and immune pathways such as *APC_co_inhibition*, *Inflammation‐promoting*, *Parainflammation*, *T_cell_costimulation*, *Type_I_IFN_Reponse*, and *Type_II_IFN_Reponse*. To investigate the differences in TMB between high‐ and low‐risk groups, we used TMB data from KIRC to calculate the mutation burden score of the samples and the number of mutations in the genes, and sorted the mutation counts in descending order. As shown in Figure 6E,F, we plotted the waterfall plots of the top 15 genes in high TMB and low TMB and conducted differential analysis. We found that the differences between high TMB and low TMB were similar. Subsequently, we analyzed the survival situation between high and low TMB, as shown in Figure 6G, and found significant differences between them. Furthermore, as shown in Figure 6H, by combining the high‐ and low‐risk joint analysis, we found that samples with high TMB and high risk had the shortest survival period, and the risk value had a higher weight in the joint analysis than TMB.

### 3.5 Performance of individualized clinical decision model

**FIGURE 7:**
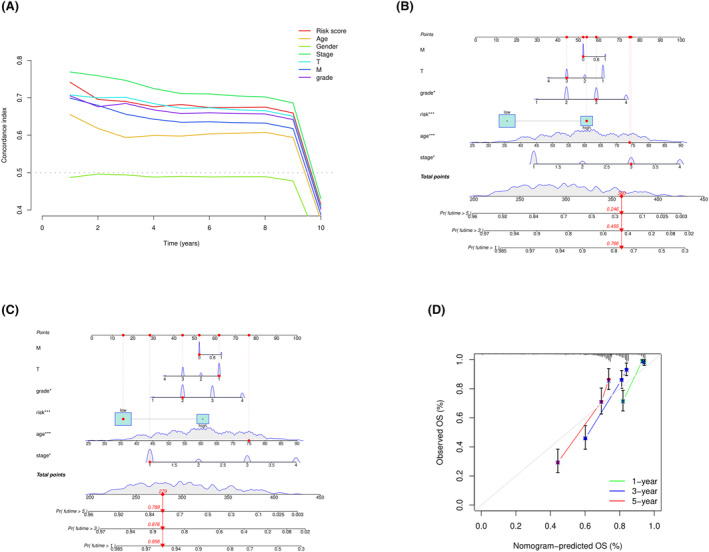
Individualized clinical decision‐making model (nomogram model). (A) shows the 0–10 year *C*‐index curve of the DNN model's predicted results (risk score) and clinical features. (B) and (C) respectively show the display of the nomogram model in high‐risk and low‐risk samples. (D) shows the calibration curves of the nomogram model at the first, third, and fifth years.

To better meet clinical needs and consider the individual differences in survival time between samples, we established a nomogram model. First, we plotted the *C*‐index curves for DNN model prediction results and clinical features over 0–10 years, as shown in Figure 7A, and found that all except gender were above 0.6. Therefore, we used DNN model prediction results and age, stage, grade, T stage, and M stage to establish the nomogram model and demonstrated the model using a high‐risk sample TCGACW‐5584 and a low‐risk sample TCGA‐BP‐5195, as shown in Figure 7B,C, respectively. In addition, as shown in Figure 7D, we evaluated the model using calibration curves for the first, third, and fifth years, and it can be seen that the accuracy of the model was good for each year.

### 3.6 Screening results for sensitive anticancer drugs

**FIGURE 8:**
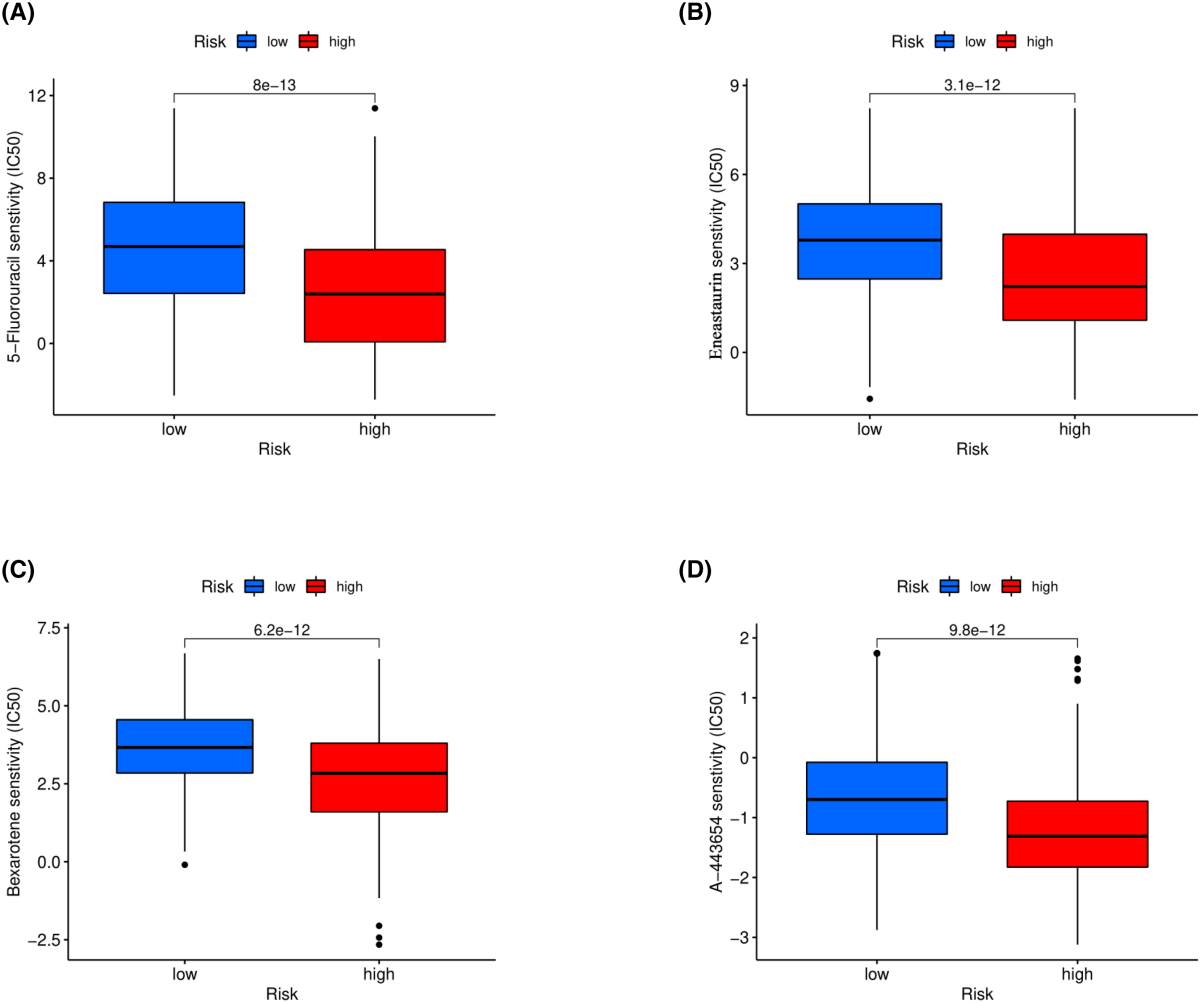
Drug screening results for KIRC. We found that (A) 5‐fluorouracil, (B) enzastaurin, (C) bexarotene, and (D) A‐443654 are highly sensitive drugs.

Through screening 251 anticancer drugs, we identified 4 top‐ranked sensitivity drugs, namely 5‐Fluorouracil, Enzastaurin, Bexarotene, and A‐443654, as shown in Figure 8A–D, respectively.

**FIGURE 9:**
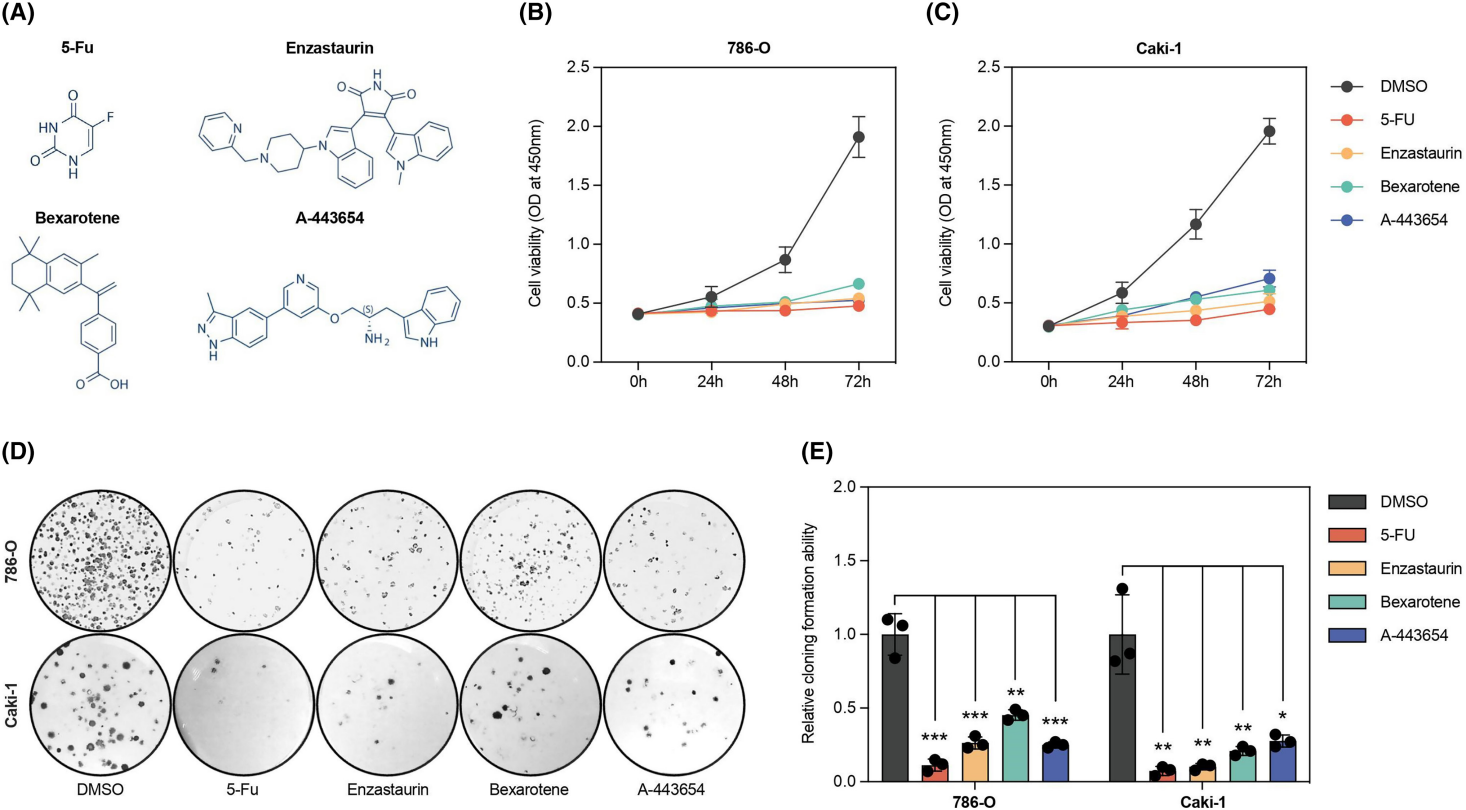
The cell experiment results of KIRC sensitive drugs. (A) shows the molecular structure of the 4 sensitive drugs. (B) and (C) display the CCK‐8 assay results of the 4 sensitive drugs on KIRC cell lines 786‐O and Caki‐1, respectively. (D) and (E) demonstrate the colony formation assay results of the 4 sensitive drugs on KIRC cell lines 786‐O and Caki‐1.

By conducting cell experiments on the aforementioned 4 sensitive drugs, it was found that all 4 drugs could inhibit the proliferation and clonogenicity of KIRC tumor cells, as shown in Figure 9. Among them, the results of the CCK‐8 assay in Figure 9B,C show that 5‐fluorouracil has the best inhibitory effect on proliferation in both 786‐O and Caki‐1 cell lines after 3 days. Additionally, as seen in Figure 9D,E 5‐fluorouracil also has the best inhibitory effect on the clonogenicity of the two KIRC cell lines.

### 3.7 Performance comparison of GNN models

**FIGURE 10:**
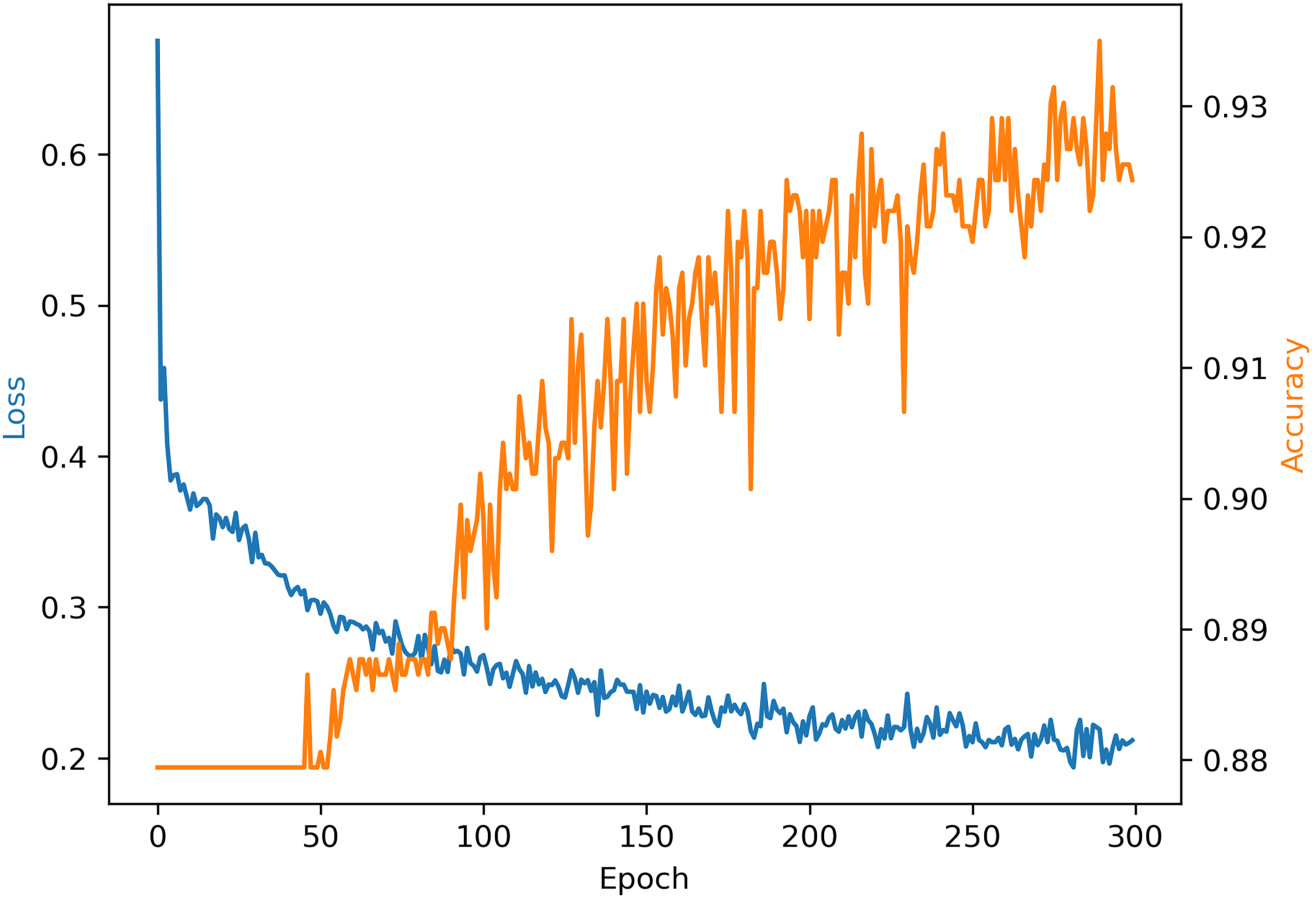
The training results of GraphSAGE.

The task of the GNN model is node classification prediction, where a prediction of 1 indicates the existence of interaction between the drug‐target pair represented by the node, and 0 indicates no interaction. The loss data and node classification accuracy of GraphSAGE are shown in Figure 10.

After repeating the training of GraphSAGE 10 times and taking the average, we found that the performance reached its optimal level at epoch = 300, with an average accuracy of 0.817 ± 0.013 on the test set at that time.

In addition, we also selected GAT^23^ and GTN^24^ as comparative models, and the results are shown in Table 1.

**TABLE 1:** Comparison of GNN models.

Models	Metrics	Epochs					
		50	100	150	200	250	300
GAT	Loss	0.58997	0.58747	0.59485	0.59200	0.59016	0.59451
	Accuracy	0.588	0.588	0.588	0.588	0.588	0.588
GTN	Loss	0.48071	0.47060	0.47799	0.46461	0.44113	0.45850
	Accuracy	0.588	0.608	0.608	0.608	0.647	0.608
GraphSAGE	Loss	0.30394	0.26681	0.23025	0.21507	0.21463	0.21184
	Accuracy	0.811	0.821	0.821	0.802	0.830	0.830

As our model needs to make predictions for new nodes, we did not conduct experiments with the transductive GCN, but instead compared it with GAT and GTN, both of which have attention mechanisms. The final experimental results show that GraphSAGE performs the best.

### 3.8 GraphSAGE‐based drug‐target prediction

We connected the four drugs selected by bioinformatics methods and 341 targets as new nodes to predict the targets that can interact with the four drugs. Table 2 lists the top 15 nodes ranked by drug‐target interaction scores.

**TABLE 2:** The top 15 nodes ranked by drug‐target interaction scores.

DrugBank ID	Drug name	Uniprot ID	Target name
DB00307	Bexarotene	P10635	Cytochrome P450 2D6
DB00544	5‐Fluorouracil	P10635	Cytochrome P450 2D6
DB06486	Enzastaurin	P10635	Cytochrome P450 2D6
DB08073	A‐443654	P10635	Cytochrome P450 2D6
DB00307	Bexarotene	O60656	UDP‐glucuronosyltransferase 1A9
DB06486	Enzastaurin	O60656	UDP‐glucuronosyltransferase 1A9
DB08073	A‐443654	O60656	UDP‐glucuronosyltransferase 1A9
DB00307	Bexarotene	P07949	Proto‐oncogene tyrosine‐protein kinase receptor Ret
DB08073	A‐443654	P07949	Proto‐oncogene tyrosine‐protein kinase receptor Ret
DB06486	Enzastaurin	P22309	UDP‐glucuronosyltransferase 1A1
DB00307	Bexarotene	P22309	UDP‐glucuronosyltransferase 1A1
DB00544	5‐Fluorouracil	O60656	UDP‐glucuronosyltransferase 1A9
DB08073	A‐443654	P21802	Fibroblast growth factor receptor 2
DB00544	5‐Fluorouracil	P07949	Proto‐oncogene tyrosine‐protein kinase receptor Ret
DB08073	A‐443654	P02768	Albumin

Among the top 15 nodes ranked by interaction scores, there are seven types of targets. It is worth mentioning that all seven types of targets are directly related to KIRC, while the 293 similar targets provided as supplementary data did not appear in this ranking, indirectly proving the rationality of our selection and training of the GNN model. Among these 15 nodes, there are 4 nodes with drug‐target interactions that have previous research reports, including six studies on the combination of 5‐fluorouracil with cytochrome P450 2D6 and UDP‐glucuronosyltransferase 1A9. Studies by Coate et al.,^25^ Rondina et al.,^26^ and Ji‐Young et al.^27^ all showed that 5‐fluorouracil has a certain inhibitory effect on cytochrome P450 2D6, while studies by Cecchin et al.,^28^ Martinez‐Balibrea et al.,^29^ and Hazama et al.^30^ demonstrated that there is an interaction between 5‐fluorouracil and UDP‐glucuronosyltransferase 1A, which has a certain therapeutic effect on colorectal cancer (CRC) patients. In addition, Volakakis et al. proved that Bexarotene has the ability to co‐regulate Proto‐oncogene tyrosineprotein kinase receptor Ret,^31^ which is consistent with our prediction results. Federica et al. found that 5‐fluorouracil can treat CRC with Proto‐oncogene tyrosine‐protein kinase receptor Ret mutations.^32^ Although most of these previous studies are related to CRC, they all focused on the drug‐target interactions, and because these targets are directly related to KIRC, it is possible that the mechanisms of interaction of these drugs and targets can be applied to KIRC treatment research in the future.

## 4 DISCUSSION

KIRC is a common tumor with a high recurrence and mortality rate.^33^ Currently, the commonly used diagnostic staging for KIRC in clinical practice is T, M, and N staging. Previous studies have shown that the 5‐year survival rates for Stages I–IV are 91%, 74%, 67%, and 32%, respectively.^34^ Although partial nephrectomy for small tumors and radical nephrectomy for large tumors remain the “gold standard” treatment methods,^35^ the long‐term treatment outcomes after surgery are poor, and the mortality and recurrence rates remain high.^36^ Therefore, the development of new prognostic markers and models is necessary.

Copper is an important trace metal element in the human body, and an appropriate amount of copper plays a crucial role in maintaining biological functions. However, excessive copper can lead to cell death.^37^ Cuproptosis has been discovered as a novel cell death mechanism. It mainly occurs during cell respiration, and activates the toxic oxidative stress within cells, leading to cell death.^38^ Recent studies have shown that copper is present in large amounts in tumor tissues of various cancers, including breast cancer,^39^ colorectal cancer,^40^ and melanoma.^41^ Therefore, cuproptosis‐related genes can be used as potential tumor markers to predict tumor progression. And among the cuproptosis‐related genes we screened, FDX1 is associated with elesclomol mediated toxicity in cancer cells, where FDX1 binds directly to elesclomol, converts copper ions to cuprous ions, and releases them in mitochondria.^10^, ^42^ In addition, genome‐wide and metabolism‐focused CRISPR screens identified FDX1 and metabolic enzymes required for lipoic acid synthesis, as well as the known lipoylated protein targets of the pyruvate dehydrogenase (PDH) complex, DLAT, PDHA1, and PDHB, as mediators of copper ionophore toxicity.^43^ The uptake of copper ions by tumor cells also requires the involvement of SLC31A1, which is a major transporter of monovalent copper ions, so that elevated and reduced levels of SLC31A1 expression also directly affect intracellular copper ion levels.^44^ ATPases, including ATP7A and ATP7B, are associated with extracellular excretion of copper and they export copper ions bound to metal‐binding sites in the presence of ATP.^45^ It is worth noting that in tumor cells, copper homeostasis is maintained by interactions between multiple proteins. For example, the movement of copper ions in and out of the cell is controlled by the copper ion transporters SLC31A1 and ATP7B. Therefore, our DNN prognostic model built from these cuproptosis‐related genes is capable of predicting the prognostic survival of KIRC patients.

Most prognostic models are based on the Cox model,^46^, ^47^, ^48^ which only performs linear calculations. However, in reality, it cannot be assumed that data completely satisfies linear relationships. Therefore, when using the Cox model for advanced relationship building, a large amount of experimental data is required. For scarce survival data with nonlinear characteristics, a single Cox model is no longer applicable. As DNN models can significantly affect the nonlinear combination of variables, we propose a DNN model based on cuproptosis‐related genes for KIRC prognosis. We selected 10 different cuproptosis‐related genes from TCGA data and constructed a DNN model. We used Bayesian optimization to fit the model to the best hyperparameters and compared the performance of the DNN model and the Cox model using the ROC curve. The results show that the DNN model has better predictive ability and the highest AUC value of the model can reach 0.739. Compared with the Cox prognostic model built by Li et al. using telomere‐related genes,^49^ the AUC value of our DNN model was 0.055 higher than the AUC value of their Cox model on the total TCGA data, and compared to the Cox model built by Ning et al. using hypoxia‐related genes,^50^ the AUC value of our model was 0.059 higher than theirs. In addition, in the Cox prognostic model established by Liu et al. using calcium‐related genes,^51^ the AUC value was only 0.664 at 1 year, which was also 0.075 lower than the AUC value of our DNN prognostic model. We also set an external validation set to evaluate the generalization ability of the DNN model, which ultimately showed good results. It is worth mentioning that our model also has significant independence. It also shows that our DNN model can independently predict the prognostic risk of a patient, and the prediction results can provide a reference for understanding the survival of a patient, so as to efficiently formulate the next treatment plan for the patient. Therefore, the prognostic prediction of our DNN model can also reduce the work pressure of doctors in prognosis to some extent. In order to better analyze KIRC samples, we conducted comprehensive differential analysis of high‐ and low‐risk groups in various aspects. In terms of clinical, there was a significant difference between the risk groups in terms of PFS and stage.

In terms of functionality, we analyzed gene pathways, immune microenvironment, and TMB in detail. In addition, to better participate in clinical decision‐making, we used DNN prediction results and clinical features (age, stage, grade, and T, M staging) to establish a nomogram model, which provided survival information for patients in the first, third, and fifth years. Finally, we analyzed 251 anticancer drugs and selected 4 highly sensitive drugs: 5‐fluorouracil, enzastaurin, bexarotene, and A‐443654. Among them, 5‐fluorouracil, as a pyrimidine uracil analog, has a good anticancer effect. It mainly affects pyrimidine synthesis by inhibiting thymidylate synthase and has been widely used in the treatment of several common malignancies, including colonic carcinoma, breast cancer, and skin cancer.^52^ Although RCC is relatively resistant to chemotherapy and immunotherapy, Gebrosky et al. found that α‐2b‐interferon and 5‐fluorouracil can still have therapeutic effects on RCC.^53^ As KIRC is a common subtype of RCC, it is highly likely to use 5‐fluorouracil for treatment, which also partially confirms our prediction results.

In addition, to further explore the target of drugs in cancer treatment, we predicted the targets corresponding to the above drugs based on the GNN model. In order to better predict drug‐target pairs beyond the training data, we used GraphSAGE, which is more suitable for inductive learning, as our prediction model, and the model achieved an accuracy of up to 0.83.

Although the current model has shown some predictive value in patient prognosis and drug‐target screening, there is still room for improvement in the predictive performance of the model. For GNN models, they need to aggregate the information of the nodes of the graph data during training to make the final prediction. However, GNNs with fewer layers cannot effectively aggregate the node information of the whole graph data, so deepening the number of layers of the GNN model will be a strategy to improve the prediction performance of the model in the future, and in the process of deepening the number of layers of the GNN model, how to solve the problem of over‐smoothing and degradation of the deeper GNN model is also something we need to think about in the future. Similarly, for DNN models, deepening the number of network layers and increasing the number of neurons can also enable them to learn deeper data feature representations, which is also the direction of DNN model optimization in the future. Therefore, we plan to continue optimizing the model and timely supplement new data to ensure the generalization ability of the model in the future. At the same time, we will also collect real‐world data to further verify the performance of the model.

## 5 CONCLUSIONS

In this study, 10 cuproptosis‐related genes were screened by differential analysis, and a DNN prognostic model was established by these genes, and the best AUC value of the model could reach 0.739. In addition, 4 highly sensitive drugs that might be used for the treatment of KIRC were screened, including 5‐fluorouracil, enzastaurin, bexarotene, and A‐443654.

Among the 15 drug‐target pairs predicted by GraphSAGE, 4 drug‐target pairs have been confirmed in previous studies, including 3 corresponding targets of 5‐fluorouracil and 1 corresponding target of bexarotene, which confirms the prediction effect of GraphSAGE on the target of KIRC high sensitivity drugs.

Overall, we replaced the traditional Cox method with the DNN model, established a prognostic model of 10 cuproptosis‐related genes, and verified the superiority of the DNN model's prediction ability. We also analyzed the differences between high‐ and low‐risk groups from multiple perspectives. For the individual differences in patient survival, we also established a nomogram model to predict survival probability. In terms of drug treatment for KIRC, we screened high‐sensitivity drugs for high‐risk patients to seek new drug treatment options, and predicted the corresponding targets of high‐sensitivity drugs through an appropriate GNN model.

## AUTHOR CONTRIBUTIONS

**Yixin Liu:** Conceptualization (lead); data curation (lead); methodology (lead); writing – original draft (lead). **Yuan Shao:** Funding acquisition (supporting); validation (lead). **Zezhou Hao:** Data curation (supporting); investigation (lead); methodology (supporting); writing – original draft (supporting). **Xuanzi Lei:** Investigation (supporting); validation (supporting). **Pengchen Liang:** Writing – review and editing (supporting). **Qing Chang:** Funding acquisition (supporting); writing – review and editing (lead). **Xianjin Wang:** Funding acquisition (lead); validation (supporting); writing – review and editing (supporting).

## FUNDING INFORMATION

This work is supported in part by funds from the Combination of Medical Care and Health Project of Shanghai University of Traditional Chinese Medicine (YYKC‐202101‐020), and the Key Projects of Jiading District Health Commission, Shanghai (no. 2020‐ZD‐03).

## CONFLICT OF INTEREST STATEMENT

The authors declare that they have no competing interests.

## ETHICS APPROVAL AND CONSENT TO PARTICIPATE

TCGA, GEO, and DrugBank belong to public databases. The patients involved in the database have obtained ethical approval. Users can download relevant data for free for research and publish relevant articles. Our study is based on open source data, so there are no ethical issues and other conflicts of interest.

## Data Availability

The datasets and codes used and/or analyzed during the current study are available from the corresponding author on reasonable request.
